# Inter-observer variability in radiotherapy contouring with the use of autocontouring software: A systematic review

**DOI:** 10.1016/j.ctro.2026.101144

**Published:** 2026-03-09

**Authors:** Polly Darby, Emily Kilgour, Chee Kin Then, Andrew Bromiley, John McLellan, Anne E. Kiltie

**Affiliations:** a Department of Radiotherapy Physics, NHS Grampian, Aberdeen, Scotland, UK; b School of Medicine, Medical Sciences, and Nutrition, University of Aberdeen, Aberdeen, Scotland, UK; c Rowett Institute, School of Medicine, Medical Sciences, and Nutrition, University of Aberdeen, Scotland, UK; d Department of Radiation Oncology, Shuang Ho Hospital, Taipei Medical University, New Taipei City, Taiwan; e Graduate Institute of Medical Sciences, College of Medicine, Taipei Medical University, Taipei, Taiwan; f Core Laboratory of Human Microbiome, Office of Research and Development, Taipei Medical University, Taipei, Taiwan; g Aberdeen Cancer Centre, University of Aberdeen, Scotland, UK; h Department of Oncology, NHS Grampian, Scotland, UK

**Keywords:** Radiotherapy, Radiotherapy planning, Computer-assisted, Observer variation, Software, Deep learning

## Abstract

Inter-observer variability (IOV) in radiotherapy contouring remains a significant source of uncertainty, especially for complex anatomical regions. Autocontouring software, including both atlas-based and deep-learning-based models, aims to improve contouring consistency and reduce workload. A systematic review was conducted in accordance with PRISMA guidelines to evaluate the impact of autocontouring software on IOV. Twenty-five eligible studies were identified that quantitatively assessed IOV using these tools. Extracted data included anatomical site, observer and case numbers, contouring method and evaluation metrics. Most studies reported significant reductions in IOV with the use of autocontouring. Edited autocontours frequently achieved mean Dice Similarity Coefficient (DSC) values above 0.85 for clinical and planning target volumes and 0.90 for organs at risk (OARs) with well-defined anatomy, using deep learning methods. Deep-learning-based models demonstrated greater consistency compared to atlas-based methods. Structures such as the lungs, heart and bladder showed the most substantial improvements, while anatomically indistinct targets such as the prostate bed and pelvic lymph nodes showed limited benefit. However, discrepancies remained between observers for certain structures despite the use of automation. Overall, autocontouring tools, particularly deep-learning models, improve contouring consistency in radiotherapy planning. However, performance is strongly influenced by anatomical complexity and segmentation method. Larger multi-institutional studies and standardised evaluation protocols are needed to support widespread clinical adoption and strengthen quality assurance.

## Highlights

• Autocontouring significantly reduces inter-observer variability in delineation.
• Deep learning segmentation outperforms atlas-based methods in consistency.
• Edited contours achieve high Dice scores for well-defined targets and OARs.
• Reduced variability for lungs, bladder, heart; limited benefit in complex anatomy.
• Clinical utility remains structure-dependent; manual review is still required.

## 1 Introduction

Radiotherapy aims to deliver a high dose of radiation to predefined volumes while sparing surrounding healthy tissue [1]. Accurate delineation of high-dose target areas and organs at risk (OARs) is essential for effective treatment planning and delivery [2], [3], [4]. Indeed, errors in identifying and contouring tumour volumes are a major source of uncertainty in radiotherapy treatment [5], and inaccurate OAR delineation has been linked to increased toxicity [6], [7].

### 1.1 Manual delineation and observer variability

Manual delineation of targets and OARs is a time-consuming task and represents a significant proportion of a clinical oncologist’s workload, particularly for complex treatment sites, such as the head and neck [8], [9]. This task is often a bottleneck in the radiotherapy workflow.

Studies indicate that contour quality is influenced by the observer’s experience, as well as by institutional and national differences in contouring standards [10], [11], [12], [13]. Numerous studies have shown substantial inter-observer variability (IOV) across different treatment sites and clinical trial contexts [3], [13], [14], [15], [16], [17], [18], [19], [20], [21], [22].

### 1.2 Autocontouring software

Autocontouring software aims to reduce contouring time and improve consistency between observers [23]. While variability in international standards presents challenges, it also highlights the need for data-driven solutions aimed at establishing consensus guidelines.

Several methodologies have been developed over the years, with varying degrees of success. Among these, only atlas-based and deep-learning-based methods have been shown to provide a fully automated solution for contouring [19], [24], [25], [26], [27], [28]. Atlas-based methods use deformable image registration to transfer contours from template datasets to new patient images. These methods can improve consistency and reduce the contouring time for specific treatment sites, such as the head and neck, prostate and lung [29], [30], [31]. However, their effectiveness is limited by the accuracy of deformable image registration and the anatomical variability of patients included in the atlas [32], [33].

In contrast, deep-learning models, trained on large datasets, have shown superior contouring accuracy and speed compared to atlas-based methods [25], [34], [35], [36], [37]. In particular, deep learning methods excel in segmenting across various patient body sizes and shapes, producing reliable results even with significant anatomical differences [38], [39]. Despite these advantages, manual editing is typically required before clinical use [40], [41], [42]. Whilst this editing step remains resource-intensive, autocontouring systems have been shown to reduce contouring time by 30–40% [43], [44], [45], [46].

With ongoing advances in AI-based autocontouring, such software is expected to become an integral component of radiotherapy treatment planning. By reducing observer dependence, these tools have the potential to shorten planning timelines, reducing the risk of tumour progression during the interval between the planning scan and the start of treatment, a particularly critical benefit for rapidly proliferating tumours. Moreover, autocontouring facilitates the incorporation of additional anatomical structures into the planning process without imposing further time constraints, thereby enhancing the feasibility of treatment planning studies and clinical trials designed to reduce radiotherapy-induced toxicity to organs we currently do not consider when planning, such as the hippocampus or specific heart structures.

This systematic literature review examines the impact of incorporating autocontouring software into the contouring workflow on contour consistency across multiple disease sites and organs, reflecting contempory clinical practice across many centres.

## 2 Methods

### 2.1 Search strategy

A systematic review of the existing literature was conducted in February 2026, following the Preferred Reporting Items for Systematic Reviews (PRISMA) guidelines. The databases searched were Embase, Medline, Cochrane Library, and Web of Science. The reference lists from the selected studies were reviewed to identify any further relevant research. The search strategy for Embase and Medline is detailed below. This was adapted for the Cochrane Library and Web of Science databases.

1. Exp Radiotherapy/
2. (radiotherapy).tw,kw.
3. (radi$ adj3 (treatment or therap$)).tw,kw.
4. 1 or 2 or 3
5. (auto$ adj3 (contour$ or delineat$ or segment$)).tw,kw.
6. (autocontour$).tw,kw.
7. (autodelineat$).tw,kw.
8. (autosegment$).tw,kw.
9. (AI adj3 (contour$ or delineat$ or segment$)).tw,kw.
10. (artificial adj3 (contour$ or delineat$ or segment$)).tw,kw.
11. (deep learning adj3 (contour$ or delineat$ or segment$)).tw,kw.
12. (machine learning adj3 (contour$ or delineat$ or segment$)).tw,kw.
13. 5 or 6 or 7 or 8 or 9 or 10 or 11 or 12
14. (Inter-observer vari$).tw,kw.
15. (Inter observer vari$).tw,kw.
16. (Interobserver vari$).tw,kw.
17. 14 or 15 or 16
18. 4 and 13 and 17

The inclusion criteria for this review were limited to studies published in peer-reviewed journals that included an assessment of the IOV of contours that were manually edited after using autocontouring software. The search focused on studies published between January 2007 and February 2026. We excluded review articles, conference abstracts, editorials and errata, animal studies, and any articles not published or translated into English.

### 2.2 Selection of studies

All references were compiled into a single database, and duplicates were manually removed. The remaining references underwent two review phases: [1] two reviewers independently screened all titles and abstracts, and [2] the selected publications were reviewed in full text by a single reviewer. After the abstract screening phase, if any conflicts arose, the abstracts in question were reassessed by both reviewers until a consensus was reached.

### 2.3 Study Quality, assessment of heterogeneity, publication bias and quality assessment

A risk of bias and applicability assessment was conducted for all studies included in the final synthesis, using an adapted Quadas 2 checklist. This checklist evaluated key domains, including patient or case selection, observer selection, autocontouring software and index test, the use of reference standard, contour editing process, metrics reported, and study design and reporting.

### 2.4 Data Extraction and assessment

The metrics used to quantify IOV were extracted from all selected studies, and these metrics were used for assessment. We also recorded the number of patients or scenarios and the number of observers used in the studies. Finally, we gathered information on the method of autocontouring, the software used for this purpose and the treatment site assessed.

## 3 Results

**Fig. 1:**
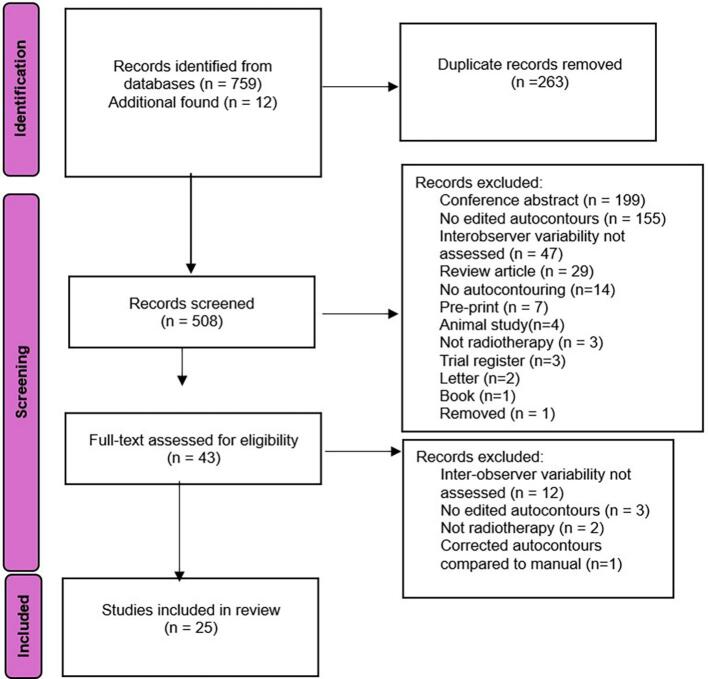
PRISMA 2020 flow chart diagram.

The results of the literature search and screening process, following PRISMA guidelines, are outlined in Fig. 1. A total of 771 records were identified: 759 from the initial database search and 12 additional articles found through reference screening. Following the identification of literature, 263 duplicates were identified via the DOI and removed from the study; 508 unique studies were screened at the title and abstract level.

Following the abstract screening phase, 465 studies were excluded. Reasons for exclusion were conference abstracts (n = 199), no edited autocontours (n = 155), no evaluation of IOV (n = 47), review articles (n = 29), autocontouring software not assessed (n = 14), pre-print (n = 7), animal study (n = 4), not radiotherapy (n = 3), trial register (n = 3), letter (n = 2), book (n = 1), and one article had been removed from publication. Fourty-three studies progressed to full-text screening, with 18 subsequently excluded; 12 did not assess IOV, three included no editing of autocontours, two were not radiotherapy related and one study compared corrected contours directly to manual contours. Twenty-five studies were included in the final synthesis.

### 3.1 Risk of bias assessment

**Fig. 2:**
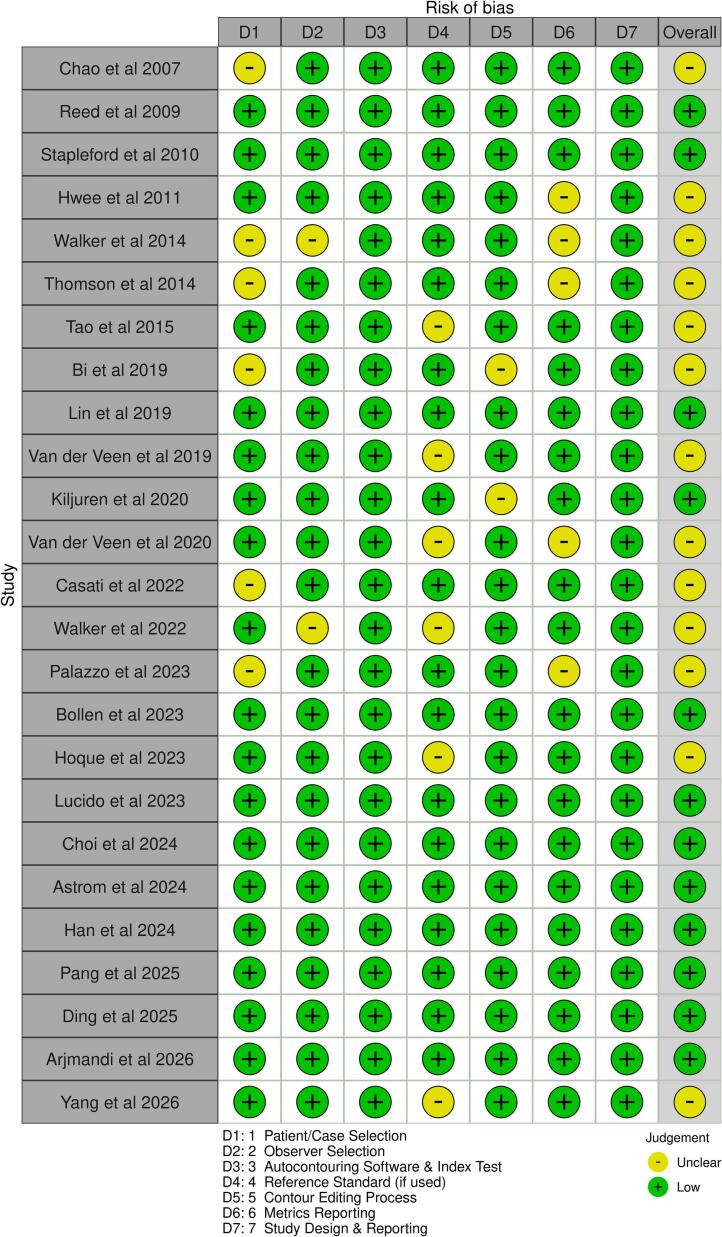
Risk of bias summary for all studies included in the final synthesis.

**Fig. 3:**
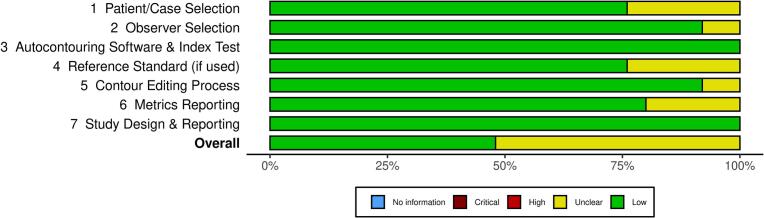
Overall assessment of risk of bias for all studies included in the final synthesis.

Of the 25 studies included, 12 were rated as low risk of bias across all domains, while 13 had some concerns in at least one domain (Fig. 2). The most common sources of potential bias were incomplete reporting of patient selection methods and the use of non-standard or institution-specific reference contours (Fig. 3). In contrast, study design, autocontouring methodology, and observer selection were generally well reported.

Several studies reused the same imaging datasets for both manual and autocontour editing tasks. Notably,. Bi et al. [47], Tao et al. [48], Van der Veen et al. [49], Lin et al. [24] and Kiljunen et al. [36] reported intervals of one month, four weeks, 15.5 days, two weeks and one week, respectively, between contouring phases. These short gaps raise the potential for recall bias, as observers may have retained memory of their initial contours, potentially inflating agreement scores and underestimating true IOV.

Despite these limitations, most studies provided adequate detail on autocontouring methods, observer credentials, and evaluation metrics.

### 3.2 Study Characteristics

A detailed summary was compiled of the included studies outlining key features such as patient or case selection, observer selection, autocontouring software used, what reference standard was used, the contour editing process, metrics report and study design (Table 1).

**Table 1:** Characteristics of included studies.

**Study**	**1. Patient/Case Selection**	**2. Structures Assessed**	**3. Observer Selection**	**4. Autocontouring Software**	**5. Reference Standard (if used)**	**6. Contour Editing Process**	**7. Metrics Reporting**	**8. Study Design & Reporting**
*Chao et al 2007*[44]	Two patients, one base-of-tongue cancer and one nasopharyngeal cancer.	Base of Tongue patient: Three CTVs, Parotids, Spinal Cord, Brainstem. Nasopharyngeal Patient: Three CTVs, Parotids, Spinal Cord, Brainstem, Optic Chiasm, Optic Nerves, Orbits.	Eight board-certified radiation oncologists.	In-house developed CAT system based on the “Demons” deformable registration algorithm.	Template contours created by an expert radiation oncologist using established guidelines. This is not a consensus or STAPLE-derived gold standard but used for volumetric comparison.	Three-step process: observers contoured from scratch, observers modified CAT-generated contours, GTVs were provided to minimise variation.	VOI and MDTA.	Not explicitly identified as retrospective or prospective, however, it appears to be retrospective. Also, not explicitly stated if single-centre or multi-centre study. Metrics were fully reported.
*Reed et al 2009*[43]	One breast cancer patient.	Whole Breast CTV.	Eight observers, seven radiation oncologists specialising in breast cancer and one senior resident.	DEF-SEG using accelerated ‘demons’ algorithm for registration [61].	Contours are defined by a multidisciplinary team using anatomical landmarks on a template patient. “Average” contour created as a consensus (voxels included in ≥ 5/8 observers contours)	Each observer first edited the DEF-SEG-generated contours, then re-contoured the case from scratch independently.	DSC, MDTA, Maximum surface-to-surface distance.	Not explicitly identified as retrospective or prospective. Also, not explicitly stated if single-centre or multi-centre study. Metrics were fully defined and reported in detail in both tables and figures.
*Stapleford et al 2010*[57]	Five Head and Neck cancer of the oropharynx or nasopharynx, selected based on having non-bulky neck nodes.	Bilateral Neck CTV.	Five experienced head and neck radiation oncologists.	HNC Atlas (Velocity Medical Systems, Atlanta, GA). Used deformable image registration to adapt the atlas to each patient's CT images.	STAPLE algorithm used to create a probabilistic “true” segmentation. Created two reference sets: STAPLE-manual from manual contours, STAPLE-AM from edited automatic contours.	Three-step process: physicians created manual contours on anonymised planning CTs, automatic contours were generated using the atlas, and physicians independently edited the automatic contours.	DSC Sensitivity, False Positive rate, mean and maximum Surface Disagreement, and Contour Volume.	Single Institution study. Not explicitly identified as retrospective or prospective. Metrics were fully defined and reported in detail in both tables and figures.
*Hwee et al 2011*[58]	Five post-prostatectomy patients planned for adjuvant/salvage radiotherapy.	Prostate Bed, Bladder, Femoral Heads, Penile Bulb, Rectum.	Five expert genitourinary radiation oncologists.	MIM (v5.2, MIMVista Corp, Cleveland, OH). Multi-atlas segmentation engine using deformable image registration, where the atlas was built using RTOG-compliant contours from 75 patients.	The STAPLE algorithm was used to generate consensus contours across observers.	Observers edited AABS-generated contours. All edits were made independently, using anonymised and blinded datasets.	DSC	Three-stage single-institution prospective design: Atlas building, observer and AABS contouring and STAPLE creation, blinded editing and evaluation. Included bias-reduction methods.
*Walker et al 2014*[6]	Forty head and neck cancer patients.	Spinal Cord, Brainstem, Chiasm, Mandible, Oral Cavity, Soft Palate, Larynx, Pharyngeal Constrictor Muscles, Optic Nerves, Parotid Glands, Submandibular Glands, Cochleae.	Eight radiation oncology residents and seven attending head and neck radiation oncologists.	SPICE AS (Philips Healthcare). A commercial atlas-based autosegmentation software.	Head and neck QA group approved contours.	Each resident was assigned to modify autocontours or draw contours manually. Attending physicians reviewed all contours and manually corrected them as necessary.	DSC	Prospective study. Not explicitly stated if single-centre or multi-centre study Results were fully reported.
*Thomson et al 2014*[59]	Ten CT scans with no artifact or tumour distortion the head and neck region.	Parotid Glands, Submandibular Glands, Larynx, Pharyngeal Constrictor Muscles, Cochleae.	Five clinicians.	Smart Probabilistic Image Contouring Engine (SPICE) software used. A commercial atlas-based and model-based automatic segmentation algorithm.	The STAPLE algorithm was used to generate consensus contours across observers.	Observers visually inspected SPICE contours and manually edited any unacceptable contours.	DSC, Mean and maximum distance to agreement.	Retrospective, single-institution study. Quantitative IOV results not fully reported.
*Tao et al 2015*[48]	Seven nasopharyngeal cancer patients with no obvious artefacts or normal tissue tumour infiltration.	Brainstem, Spinal Cord, Temporomandibular Joints, Cochleae, Temporal Lobes, Optic Nerves, Optic Chiasm, Pituitary, Mandible, Lenses, Eyes, Parotids, Thyroid, Oral Cavity, Submandibular Glands, Pharyngeal Constrictor Muscles, Supraglottic and glottic larynx.	Eight radiation oncologists from independent institutions.	ABAS (Version 2.01.00, Elekta) with multi-subject atlas. Atlas generated using STAPLE algorithm on Seven manually contoured CTs.	Multiple-subject atlas created via STAPLE from expert-contoured cases. Served as a starting point for edited contours.	Observers first did manual contouring. After a month, they independently edited ABAS contours using the same CT dataset and ABAS contours.	DSC, COV and 3D isocentre difference.	Retrospective, single institutional design. Metrics were fully reported.
*Bi et al, 2019*[47]	Nineteen patients with pN2 NSCLC receiving post-operative radiotherapy.	Primary CTV.	Eleven junior radiation oncologists from 11 institutions.	Deep Dilated Residual Network (DD-ResNet) model [59], trained on 200 cases and validated on 50.	Ground truth contours created by Three senior oncologists (>10 years’ experience) using majority voting, blinded to each other.	Each observer generated manual contours and deep learning-assisted contours. All observers used the same 19 CT image sets. All contouring tasks were completed within one month.	COV and SDD	Retrospective, single institutional design. All results were fully reported.
*Lin et al 2019*[24]	Twenty Nasopharynx patients.	Primary GTV	Eight radiation oncologists from seven centres.	Research-developed artificial intelligence tool based on VoxResNet architecture.	No consensus contour used for IOV assessment.	AI-generated contours given to each observer at least 2 weeks after manual contouring. Each observer edited the AI-generated contours where necessary. Observers were blinded to the ground truth, their own manual contours and each other’s contours.	DSC and COV.	Retrospective multicentre study. Interobserver variability results were fully and quantitatively reported.
*Van der Veen et al 2019*[49]	Fifteen head and neck patients.	Parotid Glands, Submandibular Glands, Spinal Cord, Brainstem, Larynx, Oral Cavity, Pharyngeal Constrictor Muscles, Mandible.	Two radiation oncologists.	Non-commercial convolutional neural network based deep learning auto-segmentation model.	No consensus contour used for IOV assessment.	Each observer contoured each OAR from scratch using standard institutional guidelines. Auto-generated OAR contours were then provided and each observer edited the contours as required. Observers were blinded to each others contours and an average of 15.5 days were taken between manual contouring and autocontour editing.	DSC (%), average symmetric surface distance.	Retrospective, single-centre study. All metrics were fully reported.
*Kiljuren et al 2020*[36]	Five prostate cancer patients.	Lymph Node CTV, Bladder, Prostate, Femoral Heads, Penile Bulb, Rectum, Seminal Vesicles.	Four radiation oncologists.	Mvision Delineation, a cloud-based DL model (3D U-Net and residual blocks).	No formal consensus, comparison was to manual contours drawn by clinical experts.	Each clinician reviewed and edited the same autocontoured structures using the same CT images. Editing was done independently.	DSC	Retrospective, single-centre study. Full results were reported.
*Van der Veen 2020* [51[	Twenty head and neck patients.	Elective neck clinical target volumes.	Two radiation oncologists.	Non-commercial 3D convolutional neural network based deep learning auto-segmentation model.	No consensus contour used for IOV assessment.	Each observer delineated elective neck CTVs from scratch. Observers then received auto-generated nodal CTV contours and independently reviewed and edited the contours. Each observer was blinded to each others contours. The contours generated by the two observers were reviewed and edited by a third observer if necessary.	DSC (%), HD, Mean surface distance.	Retrospective, single-centre study. Quantitative IOV results were not fully reported.
*Casati et al 2022*[8]	Six male pelvis CT scans.	Lymph Node CTV, Bladder, Rectum, Femoral Heads.	Five senior radiation oncologists.	MIM Maestro (v.6.8.2) (MIM Software Inc. Cleveland, OH). Atlas created from 55 CT's, validated and reviewed by senior radiation oncologists.	STAPLE algorithm applied to create reference contours for manual contours and reference contours set for the edited autocontours.	Each observer generated a manual and edited autocontour set. Each observer worked on the same CT images independently.	DSC and MDTA were used as metrics.	Retrospective, single-centre study. Statistical significance testing was performed.
*Walker et al 2022*[63]	Five head and neck cancer patients.	Brainstem, Larynx, Left Parotid, Left Submandibular Gland.	Four clinicians.	DLCExpert^TM^ (Mirada Medical ltd, UK). Generic head and neck model trained on CT scans using consensus guidelines.	No external gold standard was used. Inter-observer variability was assessed by comparing each observer's contours to those of the others.	Each clinician first manually contoured OARs. Then, each edited deep-learning-generated contour. Edits were performed independently for both manual and edited cases.	DSC and Distance to Agreement (DTA) were used as metrics.	Retrospective, single-centre subset within a broader multi-centre study. Statistical significance testing was performed.
*Palazzo et al 2023*[9]	Twenty intermediate/ high-risk prostate cancer patients treated with radical intent.	Bladder, Prostate, Seminal Vesicles, Rectum, Femoral Heads.	Two radiation oncologists from the same institution with > 10 years’ experience.	MIM Protégé system (v.1.1.2, MIM Software Inc. Cleveland, OH). Model trained on a multi-institutional dataset.	Manual contours based on institutional guidelines were treated as the reference for comparison.	Each observer edited the contours independently using blinded and duplicated structure sets.	DSC and HD.	Not explicitly identified as retrospective or prospective, however, it appears to be retrospective. Single-centre observational study. Results included a complete statistical report.
*Bollen et al 2023*[52]	Seventeen head and neck patients.	Primary and nodal GTV	Two radiation oncologists.	Non-commercial autocontouring software based on a 3D convolutional neural network.	No consensus contour used for IOV assessment.	Each observer manually contoured each case, blinded from each other. Each observer was then given automated contours, at least two weeks after manual delineation, and were manually corrected.	DSC (%), HD, Mean surface distance.	Retrospective, single-centre study. All results fully reported.
*Hoque et al 2023*[53]	Twenty prostate patients.	Prostate CTV, Rectum Bladder, Anal Canal and Femoral Heads.	Two radiation oncologists.	Limbus Contouring Software (version 1.0.18), AI-based deep convolutional neural network.	No consensus reference contour used for IOV analysis.	Each observer independently reviewed and edited autocontoured structures using institutional guidelines.	DSC, HD, and relative volume difference.	Retrospective, single-centre study. Results were fully reported.
*Lucido et al 2023*[62]	Nineteen head and neck patients	Brain, Lungs, Oral Cavity, Nasal Cavity, Mandible, Oesophagus, Parotid Glands, Lips, Larynx, Spinal Cord, Brainstem, Pharyngeal Constrictor Muscles, Brachial Plexus, Thyroid, Mastoid, Eyes, Submandibular Glands, Cricopharyngeal Inlet, External Auditory Canals, Retina, Lenses, Cochleae, Pituitary, Cervical Oesophagus, Lacrimal Glands, Semicircular Canals, Optic Nerves	Eight radiation oncologists and an unknown number of medical dosimetry assistants.	Non-commercial deep-learning model based on 3D U-Net.	Institutional gold standard created by two expert head and neck radiation oncologists.	Two workflows: (1) manual contours created by dosimetry assistants and revised by a radiation oncologist (2) autosegmentations revised by a radiation oncologist. All revisions were performed blinded.	DSC, HD, Added Path Length, Surface DSC.	Retrospective, single-centre study. All results fully reported.
*Choi et al 2024*[55]	Two left-sided breast cases.	Primary CTV, Lymph Nodes CTVs, Heart, Contralateral Breast, Oesophagus, Spinal Cord, Right and Left Lungs, Left Anterior Descending Artery.	Thirty radiation oncologists from independent institutions.	In-house deep learning model [58], which had been used in clinical practice since 2020, and was tested on internal/external cohorts.	A reference contour was created by a radiation oncologist and finalised by an independent expert panel of three radiation oncologists.	All observers started from identical autocontours, and all editing was conducted independently (six-month gap between phases).	DSC SDSC, HD and Added Path Length.	Retrospective, multi-centre study. Results were fully reported.
*Astrom et al 2024*[65]	Ten bladder cancer patients.	Bladder CTV	Seven radiotherapy technicians	Ethos Therapy v1.1 (Varian, Siemens Healthineers).	A ground truth contour was delineated by a senior oncologist and reviewed by an experienced medical physicist.	AI-generated contours manually edited by each observer.	Generalised Conformity Index and COV	Retrospective, single-centre study. Results were fully reported.
*Han et al 2024*[50]	Fifty-five lung cancer patients.	CTV, Lungs, Heart, Oesophagus, Liver, Spinal Cord.	Three radiation oncology residents from three institutions.	Non-commercial deep dilated convolutional neural network algorithm.	Consensus contours were produced by 3 senior radiation oncologists.	AI-generated contours were independently edited by each junior oncologist.	COV	Prospective, multi-centre study. Quantitative results fully reported.
*Pang et al 2025*[60]	Seven patients.	Parotid Glands, Submandibular Glands, Brachial Plexus, Constrictor Muscle, Lymph node levels.	Seven centres. Experience of observers not stated.	Mvision, a deep learning-based 3D encoder-decoder U-Net.	STAPLE concensus contour used generated from seven manual contours.	Each clinician manually contoured one scan and edited the autocontours of another.	DSC and HD.	Retrospective, multi-centre study. Results were fully reported.
*Ding et al 2025*[61]	Ten paediatric patients with renal tumours.	Heart, Spleen, Liver, Lungs, Kidneys, Pancreas, Stomach-bowel.	Twelve paediatric radiation oncologists from twelve different centres and nine different countries.	ProKnow v2.0.2.0 deep learning-based auto-contouring model.	The STAPLE algorithm was used to generate consensus contours across observers.	All observers started from the same auto-generated contours and edited these contours independently.	DSC, HD, Mean Surface Distance.	Retrospective multicentre and multinational study. Results included a complete statistical report.
*Arjmandi et al 2026*[54]	Fifteen prostate patients.	CTV, Rectum, Bladder, Femoral Heads.	Two radiation oncologists.	Limbus Contouring Software (V1.7.0-B3).	A reference contour was created as a consensus delineation by three experts.	All observers created manual contours then started from the same auto-generated contours and edited these contours independently. A two-month gap was given between the manual contouring and autocontour editing.	DSC and HD.	Retrospective, single-centre study with explicit assessment of automation bias. All results were fully reported.
*Yang et al 2026*[64]	Five patients with gynaecological malignancies undergoing CT-based brachytherapy.	Bladder, Rectum, Sigmoid Colon, Bowel.	Four clinicians.	Pre-clinical release of Limbus AI-based autocontouring software (v1.8).	Expert contours were used as a comparison benchmark. No specific reference contour was used.	All observers started from the same auto-generated contours and edited these contours independently.	DSC, MDTA, HD	Retrospective, single-centre study. Results were fully reported.

Abbreviations: DSC: Dice Similarity Coefficient, SDSC: Surface Dice Similarity Coefficient, HD: Hausdorff Distance, MDTA: Mean Distance to Agreement, COV: Coefficient of Variation, SDD: Standard Distance Deviation, VOI: Volume Overlap Index.

Of the studies included in the final synthesis, earlier studies predominantly focused on atlas-based approaches (n = 7) while more recent work increasingly focussed on deep-learning-based autocontouring methods (n = 18). Noting the publication dates, this distribution reflects the evolution of autocontouring technology from early atlas-driven models to more sophisticated deep-learning systems.

The number of patients or scenarios used in each study varied markedly, from a single case in Reed et al. [43] to 55 cases used in Han et al. [50]. The number of observers also varied substantially across studies, with several investigations including only two observers (Van der Veen et al. [49], Van der Veen et al. [51], Palazzo et al. [9], Bollen et al. [52], Hoque et al. [53], and Arjmandi et al. [54]), compared to a maximum of 30 observers in Choi et al [55].

Studies addressed a range of treatment sites, with head and neck being the most common (n = 12), encompassing both organs at risk and target volumes. Several studies included CTV and GTV delineation, including nodal volumes in the head and neck, breast CTV’s and pelvic lymph node CTV’s. Other anatomical sites included prostate, male and female pelvis, bladder, lung and paediatric abdominal organs.

The majority of studies employed a two-phase contouring workflow, in which observers first performed manual contouring, followed by the editing of autocontours. In general, studies explicitly stated that observers were blinded from each other, identical contours were provided and washout periods were employed to reduce recall bias. However, an explicit assessment of automation bias was only included with the study by Arjmandi et al. [54].

Studies were predominantly single-centre (n = 17), although an increasing number of multi-centre studies have emerged in recent years (n = 5). Three studies did not report if they were single or multi-centre.

### 3.2 Assessment metrics

The Dice Similarity Coefficient (DSC) was the most commonly reported metric for evaluating IOV, used in 21 of the 25 studies. This is consistent with the widespread use of this metric in Radiotherapy to evaluate spatial overlap between structures [56]. While effective, DSC is best suited to pairwise comparison between volumes, and its application in multi-observer studies requires the use of consensus or reference contours.

Several studies (n = 8), particularly earlier studies, used the Simultaneous Truth and Performance Level Estimation (STAPLE) algorithm to generate probabilistic consensus contours for DSC calculation (Stapleford et al. [57], Hwee et al. [58], Thomson et al. [59], Tao et al [48], Casati et al. [8], Pang et al. [60] and Ding et al. [61]). Others, such as Reed et al. [43], Lucido et al. [62], Choi et al. [55] and Arjmandi et al. [54], used consensus contours derived through manual agreement or averaging. Walker et al. [63] performed pairwise comparisons without a defined reference. Palazzo et al. [9], which included only two observers, relied on direct pairwise DSC comparisons.

In addition to DSC, the Mean Distance to Agreement (MDTA) was reported as a complementary metric in six studies, predominantly in earlier publications. In contrast, nine studies published from 2020 onwards reported HD, either in its standard form or as a robust variant, as an additional measure of interobserver variability. Due to the wide range of metrics used in these studies and their reporting, a *meta*-analysis was not feasible.

### 3.3 Inter-observer variability outcomes

Overall, the evidence supports the use of autocontouring software to reduce IOV for several anatomical structures (Table 2). The most consistent improvements were observed for structures with well-defined anatomical boundaries, including the lungs, femoral heads, bladder, and heart. High interobserver DSC values (≥0.92, with the majority ≥ 0.97) for these structures were reported across multiple studies, including Hwee et al. [58], Palazzo et al. [9], Hoque et al. [53], Choi et al. [55], Arjmandi et al. [54], Ding et al. [61] and Yang et al. [64].

**Table 2:** Overview of Dice Similarity Coefficient (DSC) values from all studies where it was used for three situations: between edited autocontours from different observers, between edited contours and a consensus volume, and between observers without the use of autocontouring.

Study	Structure	DSC (±SD)		
		With Autocontouring (Interobserver)	With Autocontouring (Consensus)	Without Autocontouring
*Reed et al 2009*[43]	Breast		0.94	0.92
*Stapleford et al 2010*[57]	Nodal CTV		0.89	0.79
*Hwee et al 2011*[58]	Penile Bulb		0.54 ± 0.21	0.55 ± 0.22
	Prostate Bed		0.67 ± 0.19	0.65 ± 0.14
	Bladder		0.88 ± 0.13	0.94 ± 0.03
	Left Femoral Head		0.93 ± 0.01	0.76 ± 0.26
	Right Femoral Head		0.92 ± 0.01	0.77 ± 0.23
	Rectum		0.78 ± 0.12	0.83 ± 0.07
*Walker et al 2014*[6]	Brainstem		0.99 ± 0.01	0.95 ± 0.14
	Spinal Cord		0.97 ± 0.06	0.90 ± 0.18
	Optic Nerves		0.77 ± 0.24	0.77 ± 0.24
	Optic Chiasm		0.93 ± 0.17	0.96 ± 0.14
	Mandible		0.99 ± 0.01	0.98 ± 0.05
	Parotid Glands		0.90 ± 0.11	0.90 ± 0.11
	Submandibular Glands		0.76 ± 0.26	0.77 ± 0.26
	Oral Cavity		0.87 ± 0.24	0.92 ± 0.18
	Soft Palate		0.96 ± 0.08	0.93 ± 0.14
	Cochleae		0.61 ± 0.39	0.6 ± 0.40
	Larynx		0.94 ± 0.13	0.94 ± 0.15
	Pharyngeal Constrictors		0.91 ± 0.04	0.91 ± 0.17
*Tao et al 2015*[48]	Brainstem	0.86 ± 0.04		0.83 ± 0.03
	Spinal Cord	0.82 ± 0.04		0.77 ± 0.04
	TMJ_L	0.69 ± 0.07		0.49 ± 0.18
	TMJ_R	0.71 ± 0.07		0.50 ± 0.18
	Cochlea_L	0.43 ± 0.12		0.37 ± 0.10
	Cochlea_R	0.42 ± 0.11		0.36 ± 0.11
	PCM_S	0.63 ± 0.09		0.44 ± 0.07
	PCM_M	0.64 ± 0.07		0.50 ± 0.08
	PCM_I	0.65 ± 0.06		0.50 ± 0.09
	Larynx_supraglottic	0.73 ± 0.04		0.60 ± 0.05
	Larynx_glottic	0.64 ± 0.08		0.49 ± 0.09
*Lin et al 2019*[24]	Primary GTV	0.80		0.70
*Walker et al 2022*[63]	Brainstem	0.93 ± 0.01		0.78 ± 0.03
	Left Parotid Gland	0.92 ± 0.02		0.79 ± 0.02
	Left Submandibular Gland	0.89 ± 0.04		0.80 ± 0.02
	Larynx	0.83 ± 0.01		0.84 ± 0.01
*Palazzo et al 2023*[9]	Bladder	0.98		0.93
	Prostate	0.88		0.83
	Seminal Vesicles	0.83		0.73
	Rectum	0.96		0.81
	Femoral Heads	0.97		0.65
*Hoque et al 2023*[53]	Prostate PTV	0.91 ± 0.06		
	Rectum	0.97 ± 0.03		
	Bladder	0.98 ± 0.01		
	Anal Canal	0.91 ± 0.05		
	Femur Left	0.98 ± 0.02		
	Femur Right	0.98 ± 0.03		
*Lucido et al 2023*[62]	Brain		0.99 ± 0.00	0.99 ± 0.00
	Left Lung		0.98 ± 0.00	0.98 ± 0.00
	Right Lung		0.98 ± 0.00	0.98 ± 0.00
	Oral Cavity		0.92 ± 0.04	0.86 ± 0.05
	Nasal Cavity		0.94 ± 0.01	0.86 ± 0.04
	Mandible		0.96 ± 0.01	0.95 ± 0.01
	Oesophagus		0.86 ± 0.02	0.80 ± 0.03
	Left Parotid		0.89 ± 0.01	0.86 ± 0.01
	Right Parotid		0.89 ± 0.01	0.85 ± 0.02
	Lips		0.83 ± 0.02	0.74 ± 0.02
	Larynx		0.89 ± 0.04	0.80 ± 0.04
	Spinal Cord		0.85 ± 0.01	0.75 ± 0.05
	Brainstem		0.89 ± 0.01	0.87 ± 0.01
	Pharyngeal Constrictor Muscles		0.73 ± 0.05	0.59 ± 0.04
	Left Brachial Plexus		0.71 ± 0.06	0.49 ± 0.08
	Right Brachial Plexus		0.71 ± 0.06	0.48 ± 0.06
	Thyroid		0.91 ± 0.01	0.87 ± 0.02
	Left Mastoid		0.89 ± 0.07	0.85 ± 0.06
	Right Mastoid		0.88 ± 0.06	0.86 ± 0.04
	Left Eye		0.95 ± 0.01	0.93 ± 0.01
	Right Eye		0.95 ± 0.01	0.93 ± 0.02
	Left Submandibular Gland		0.89 ± 0.02	0.87 ± 0.03
	Right Submandibular Gland		0.90 ± 0.01	0.87 ± 0.03
	Cricopharyngeal Inlet		0.87 ± 0.01	0.75 ± 0.06
	Left External Auditory Canal		0.86 ± 0.03	0.71 ± 0.04
	Right External Auditory Canal		0.87 ± 0.03	0.70 ± 0.04
	Left Retina		0.79 ± 0.02	0.72 ± 0.05
	Right Retina		0.78 ± 0.02	0.69 ± 0.05
	Cervical Oesophagus		0.81 ± 0.08	0.70 ± 0.08
	Left Lacrimal		0.71 ± 0.06	0.55 ± 0.06
	Right Lacrimal		0.71 ± 0.05	0.55 ± 0.06
	Left Semi-circular Canal		0.79 ± 0.07	0.59 ± 0.09
	Right Semi-circular Canal		0.80 ± 0.06	0.59 ± 0.09
	Left Optic Nerve		0.83 ± 0.02	0.76 ± 0.02
	Right Optic Nerve		0.85 ± 0.02	0.78 ± 0.03
	Pituitary		0.83 ± 0.05	0.71 ± 0.06
	Left Cochlea		0.87 ± 0.02	0.79 ± 0.03
	Right Cochlea		0.86 ± 0.02	0.76 ± 0.05
	Left Lens		0.85 ± 0.03	0.83 ± 0.04
	Right Lens		0.86 ± 0.02	0.85 ± 0.02
*Choi et al 2024*[55]	CTVn_L1	0.67 ± 0.22	0.73 ± 0.17	
	CTVn_L2	0.7 ± 0.25	0.65 ± 0.21	
	CTVn_L3	0.55 ± 0.23	0.61 ± 0.120	
	CTVn_IMN	0.61 ± 0.16	0.64 ± 0.15	
	CTVn_SCL	0.62 ± 0.2	0.32 ± 0.13	
	CTVp_breast	0.8 ± 0.13	0.81 ± 0.14	
	Heart	0.95 ± 0.03	0.95 ± 0.02	
	Contralateral breast	0.89 ± 0.10	0.92 ± 0.08	
	Thyroid	0.79 ± 0.12	0.82 ± 0.08	
	Oesophagus	0.81 ± 0.07	0.83 ± 0.04	
	Spinal Cord	0.79 ± 0.14	0.82 ± 0.07	
	Lung R	0.98 ± 0.01	0.98 ± 0.01	
	Lung L	0.98 ± 0.02	0.98 ± 0.01	
	Left Anterior Descending Artery	0.61 ± 0.18	0.59 ± 0.01	
*Pang et al 2025*[60]	Left Brachial Plexus	0.89 ± 0.08		0.33 ± 0.13
	Right Brachial Plexus	0.88 ± 0.08		0.33 ± 0.12
	Left Submandibular Gland	0.92 ± 0.06		0.73 ± 0.23
	Right Submandibular Gland	0.92 ± 0.06		0.72 ± 0.24
	LN_Neck_ⅠA	0.77 ± 0.20		0.49 ± 0.23
	LN_Neck_ⅠB_L	0.90 ± 0.05		0.59 ± 0.18
	LN_Neck_ⅠB_R	0.87 ± 0.07		0.59 ± 0.19
	LN_Neck_ⅠⅠⅠ_L	0.89 ± 0.08		0.70 ± 0.09
	LN_Neck_ⅠⅠⅠ_R	0.91 ± 0.07		0.68 ± 0.14
	LN_Neck_ⅠⅠ_L	0.91 ± 0.04		0.74 ± 0.08
	LN_Neck_ⅠⅠ_R	0.92 ± 0.05		0.76 ± 0.07
	LN_Neck_ⅠVA_L	0.83 ± 0.12		0.58 ± 0.14
	LN_Neck_ⅠVA_R	0.85 ± 0.11		0.59 ± 0.12
	LN_Neck_ⅠVB_L	0.75 ± 0.13		0.50 ± 0.14
	LN_Neck_ⅠVB_R	0.72 ± 0.14		0.44 ± 0.16
	Constrictor Muscles	0.85 ± 0.16		0.50 ± 0.10
	Left Parotid	0.97 ± 0.02		0.80 ± 0.08
	Right Parotid	0.97 ± 0.02		0.78 ± 0.10
*Ding et al 2025*[61]	Heart	0.95 ± 0.01		0.91 ± 0.03
	Spleen	0.95 ± 0.02		0.93 ± 0.02
	Kidney	0.96 ± 0.01		0.96 ± 0.01
	Liver	0.97 ± 0.0		0.95 ± 0.01
	Left Lung	0.96 ± 0.01		0.94 ± 0.01
	Right Lung	0.97 ± 0.0		0.94 ± 0.02
	Pancreas	0.78 ± 0.07		0.63 ± 0.23
	Stomach-Bowel	0.89 ± 0.04		0.88 ± 0.06
*Arjmandi et al 2026*[54]	CTV	0.89 ± 0.09		0.78 ± 0.11
	Rectum	0.90 ± 0.05		0.76 ± 0.11
	Bladder	0.99 ± 0.02		0.91 ± 0.03
	Right Femoral Head	0.97 ± 0.01		0.88 ± 0.01
	Left Femoral Head	0.98 ± 0.01		0.87 ± 0.01
*Yang et al 2026*[64]	Bladder	0.97		0.89
	Bowel	0.83		0.66
	Rectum	0.98		0.64
	Sigmoid	0.94		0.52

However, the magnitude of benefit varied substantially across anatomical sites and studies. Structures with poorly defined borders or greater anatomical complexity such as the prostate bed, penile bulb, pharyngeal constrictor muscles, cochleae and breast lymph node levels demonstrated lower DSC values, even with autocontouring assistance. For example, Hwee et al. [58] reported poor performance of their autocontouring tool for the prostate bed and penile bulb, while Casati et al. [8] and Choi et al. [55] found minimal benefit for pelvic and breast lymph node contours. These findings suggest that autocontouring effectiveness is highly dependent on both the anatomical structure and the algorithm used.

Importantly, none of the included studies demonstrated complete elimination of IOV. Residual discrepancies persisted across all anatomical regions, particularly for structures with poorly defined boundaries or higher subjectivity in interpretation. While most studies provided quantitative assessments of IOV, incomplete or inconsistent reporting in some cases limited direct comparison across studies. Collectively, these findings indicate that while autocontouring improves contouring consistency, it does not obviate the need for expert review and manual refinement.

## 4 Discussion

This systematic literature review assessed the impact of autocontouring software on IOV in radiotherapy contouring, providing insight into its performance within routine clinical practice.

The evidence consistently demonstrates that autocontouring tools improve delineation consistency. Nearly all included studies reported higher overlap, measured by using DSC, for edited autocontours compared to manual contours. For example, Palazzo et al. [9] found that edited contours of prostate CTVs achieved DSCs of 0.83–0.88 versus 0.73–0.83 for manual contours. Similarly, Casati et al. [8] reported that atlas-based autocontouring approximately halved IOV for several pelvic structures.

However, greater consistency does not guarantee greater accuracy. As Chao et al. [44] highlighted, improved agreement among observers may still reflect systematic errors. This underscores the importance of rigorous commissioning and quality assurance to prevent propagation of model biases.

While the overall findings support the use of autocontouring for both OARs and target volumes, several limitations should be acknowledged. Many of the included studies had narrow inclusion criteria or were conducted at a single institution, which may limit the generalisability of the results. For example, Choi et al. [55] focused exclusively on Korean institutions, and studies by Palazzo et al. [9], Hwee et al. [58] and Stapleford et al. [57] were just a few of the studies conducted at a single centre. These factors may underestimate the extent of IOV that would occur in more diverse, multi-institutional settings.

Autocontouring methods and workflows also varied widely. Earlier studies used atlas-based systems, while more recent work (2019–2026) employed deep-learning models (Table 1). This transition is promising: studies using deep learning reported very high DSCs (≈0.95–0.98) for well-defined OARs (e.g. lungs, heart) [55]. However, even these models required manual refinement to achieve clinically acceptable contours.

A key finding is that autocontouring performance is structure-dependent. Well-defined high-contrast structures, such as the bladder, lungs and femoral heads, benefited most from automation (Table 2). In contrast, autocontouring was less effective for ambiguous or highly variable structures such as the prostate bed, penile bulb, pharyngeal constrictor muscles, cochleae and breast lymph nodes, as reported by Hwee et al. [58], Tao et al. [48], Lucido et al. [62] and Choi et al.[55]. These results suggest that clinicians should exercise caution when relying on autocontouring for low-contrast or highly variable anatomy. Additional model training or atlas enhancement may be required in such cases.

Comparability across studies was further complicated by methodological heterogeneity. Reference standards, contouring workflows, and outcome metrics varied substantially. While DSC was used in 19 of the 23 studies, other metrics such as Hausdorff Distance, Added Path Length and Coefficient of Variation were reported inconsistently. This variability highlights the need for standardised evaluation protocols to facilitate benchmarking across tools and institutions.

From a clinical perspective, the results are encouraging. Autocontouring can reduce planning time and improve inter-observer agreement. For example, Palazzo et al. [9] concluded that expert-edited contours can replace manual contouring, significantly improving consistency and efficiency. Likewise, Casati et al. [8] noted improvements in reproducibility and time savings. These findings support broader adoption of validated autocontouring software to enhance treatment planning quality and throughput.

However, before widespread clinical integration, further research is required. Multi-centre validation studies are essential to assess generalisability and institutional variability. The development of benchmark datasets and shared evaluation frameworks would allow direct comparison between algorithms. As deep learning continues to evolve, new models should be tested against established ones using the same datasets to quantify residual IOV. Finally, quality assurance processes must remain central, with routine monitoring and retraining to prevent performance drift.

## 5 Conclusion

In summary, this review indicates that autocontouring software, especially modern AI-driven methods, markedly reduces IOV compared to fully manual delineation. Across multiple disease sites and organs, edited autocontours showed higher consistency. These findings underscore the growing importance of these tools for standardising radiotherapy planning. Nonetheless, existing studies are limited by heterogeneity and scale, making it difficult to quantify the exact residual IOV for each method. There is a clear need for more large-scale, multi-institutional trials and the establishment of benchmarking datasets to compare different autocontouring solutions and to guide best practice. In the meantime, clinics implementing autocontouring should rigorously commission their systems and be aware of which structures benefit most and least from automation. Future research should focus on expanding training datasets, especially for challenging targets, harmonising evaluation metrics, and investigating the clinical impact of any remaining contour discrepancies. By addressing these needs, autocontouring can fulfil its promise of making radiotherapy more consistent, efficient and accurate.

## Grant information

This research was supported by the Chief Scientist Office under grant number IFA312PD. The funder had no direct role in the preparation of this article and the decision to submit for publication.

## CRediT authorship contribution statement

**Polly Darby:** Data curation, Formal analysis, Investigation, Methodology, Project administration, Writing – original draft, Writing – review & editing. **Emily Kilgour:** Data curation, Formal analysis, Investigation, Methodology, Project administration, Writing – review & editing. **Chee Kin Then:** Methodology, Writing – review & editing. **Andrew Bromiley:** Supervision, Writing – review & editing. **John McLellan:** Supervision, Writing – review & editing. **Anne E. Kiltie:** Supervision, Writing – review & editing.

## Declaration of competing interest

The authors declare that they have no known competing financial interests or personal relationships that could have appeared to influence the work reported in this paper.
